# Trends in *Streptococcus pneumoniae* Antimicrobial Resistance in US Children: A Multicenter Evaluation

**DOI:** 10.1093/ofid/ofad098

**Published:** 2023-03-07

**Authors:** Salini Mohanty, Kristen Feemster, Kalvin C Yu, Janet A Watts, Vikas Gupta

**Affiliations:** Center for Observational and Real World Evidence (CORE), Merck & Co, Inc, Rahway, New Jersey, USA; Center for Observational and Real World Evidence (CORE), Merck & Co, Inc, Rahway, New Jersey, USA; Becton, Dickinson & Company, Franklin Lakes, New Jersey, USA; Becton, Dickinson & Company, Franklin Lakes, New Jersey, USA; Becton, Dickinson & Company, Franklin Lakes, New Jersey, USA

**Keywords:** antibiotic resistance, children, pneumococcal disease, invasive pneumococcal vaccines, *Streptococcus pneumoniae*

## Abstract

**Background:**

Antimicrobial resistance (AMR) poses a significant challenge for treating pneumococcal disease. This study assessed AMR trends in *Streptococcus pneumoniae* from US children.

**Methods:**

We evaluated antibiotic resistance, defined as facility antimicrobial susceptibility reports of intermediate/resistant, in 30-day nonduplicate *S pneumoniae* isolates from children (<18 years of age) with invasive (blood or cerebrospinal fluid/neurological) or noninvasive (respiratory or ear/nose/throat) isolates at 219 US hospital inpatient/outpatient settings in the BD Insights Research Database (January 2011–February 2020). We used descriptive statistics to characterize the percentage of antimicrobial-resistant isolates and generalized estimating equations to assess variations in resistance over time.

**Results:**

Of 7605 *S pneumoniae* isolates analyzed, 6641 (87.3%) were from noninvasive sources. Resistance rates were higher in noninvasive versus invasive isolates. Isolates showed high observed rates of resistance to ≥1 drug class (56.8%), ≥2 drug classes (30.7%), macrolides (39.9%), and penicillin (39.6%) and significant annual increases in resistance to ≥1 drug class (+0.9%), ≥2 drug classes (+1.8%), and macrolides (+5.0%).

**Conclusions:**

Among US children over the last decade, *S pneumoniae* isolates showed persistently high rates of resistance to antibiotics and significant increases in ≥1 drug class, ≥2 drug classes, and macrolide resistance rates. Efforts to address AMR in *S pneumoniae* may require vaccines targeting resistant serotypes and antimicrobial stewardship efforts.

The introduction of pneumococcal conjugate vaccines (PCVs) against *Streptococcus pneumoniae* infections transformed the epidemiology of pneumococcal disease (PD) and associated health outcomes in both children and adults [1, 2]. Initial decreases in PD rates following introduction of PCVs were accompanied by reductions in cases of antimicrobial-resistant invasive pneumococcal disease (IPD) in children in the United States (US) and globally [3–7]. PCVs can reduce antimicrobial resistance (AMR) directly by reducing the incidence of resistant pneumococcal infections as well as indirectly by reducing antibiotic use, thereby relieving selective pressure on antibiotic-resistant strains [8, 9]. However, increased AMR over time has been observed in nonvaccine serotypes in pediatric IPD [10] and in both vaccine and nonvaccine serotypes in noninvasive PD pediatric samples [11].

Currently, the Centers for Disease Control and Prevention (CDC) estimates that approximately 30% of IPD cases are caused by *S pneumoniae* isolates resistant to 1 or more clinically relevant antibiotics. Drug-resistant *S pneumoniae* isolates caused an estimated 900 000 infections and 3600 deaths per year in all age groups in 2014 and has been designated a serious threat by the CDC [12]. Worldwide, *S pneumoniae* is the fourth leading pathogen in terms of deaths associated with or attributable to resistance [13]. Current data on AMR in *S pneumoniae* are essential not only for clinical management, but also to assess evolutionary AMR trends in this important pathogen and prioritize efforts to reduce resistance. However, there is a lack of information on *S pneumoniae* resistance in the pediatric population, which does not have as many treatment options as adult patients [14]. In particular, there is a dearth of information on resistance in pediatric noninvasive PD infections, which constitute the majority of PD cases [2]. We therefore evaluated AMR in *S pneumoniae* isolates obtained from children with invasive or noninvasive PD.

## METHODS

### Study Design

This was a retrospective study of antibiotic susceptibility of specified nonduplicate *S pneumoniae* isolates (first noncontaminant *S pneumoniae* isolate within 30 days) collected from hospitalized and ambulatory pediatric patients (aged <18 years at time of culture collection) between January 2011 and February 2020 as part of routine clinical management. IPD cases were defined as those with *S pneumoniae* isolates obtained from cerebrospinal fluid (CSF)/neurology samples or blood (including valve and ventricle catheter tip sources). Noninvasive PD cases were defined as those with *S pneumoniae* isolates obtained from respiratory samples or the ear, nose, or throat (ENT). Skin/wound, urine, and other nonsterile sources not listed above were evaluated but excluded from statistical modeling as they are not commonly associated with *S pneumoniae* infections. Cultures likely to be associated with contamination or colonization, such as environmental/surveillance specimens from rectal or nasal swabs, were excluded by use of a previously described algorithm [15].

Reporting institutions consisted of US hospitals in the BD Insights Research Database (Becton, Dickinson and Company, Franklin Lakes, New Jersey), which includes small and large hospitals in urban and rural areas and provides geographical representation across the US [16, 17]. The study was approved as involving use of a limited retrospective data set for an epidemiology study and deemed exempt from consent by the New England Institutional Review Board/Human Subjects Research Committee (Wellesley, Massachusetts) and conducted in compliance with Health Insurance Portability and Accountability Act requirements.

AMR was evaluated in *S pneumoniae* isolates based on facility reports; minimal inhibitory concentration (MIC) breakpoints were not standardized across facilities. Antibiotic resistance was assessed using the following definitions:

- Penicillin resistant: Intermediate (I) or resistant (R) to penicillin.
- Macrolide resistant: I or R to erythromycin, azithromycin, or clarithromycin.
- Fluoroquinolone resistant: I or R to levofloxacin or moxifloxacin.
- Extended-spectrum cephalosporin (ESC) resistant: I or R to ceftriaxone, cefotaxime, or cefepime.
- Tetracycline resistant: I or R to doxycycline or tetracycline.
- Any drug class or ≥2 drug class resistant: I or R to any of the tested antibiotics or ≥2 of the drug classes listed above, respectively.

### Outcomes

For each category of resistance defined above, we evaluated the percentage of resistance (mean number of resistant isolates per total isolates tested) overall, by invasive versus noninvasive PD, and by year, sex, age group, and hospital characteristics.

### Statistical Analysis

Descriptive statistics of the percentage of resistant isolates over time were presented by cross-tabulation. For the multivariate analyses, the generalized estimating equation (GEE) method and logistic regression with a first order autoregressive variance-covariance matrix was used to evaluate the quarterly trends of percentage AMR. In the GEE framework, the time series data (count of resistant isolates) were viewed as repeated measures and hospital/facility modeled as random effect. All analyses were also stratified by invasive and noninvasive source type. Key additional factors included in the analyses were setting of isolate collection (ambulatory, admission period [first 3 days of admission, with day of admission considered day 1], and hospital-onset period [>3 days postadmission]), age group, sex, and source type (blood, CSF/neurological, respiratory, and ENT). Multivariate analyses were controlled for hospital demographics (bed size, urban/rural location, teaching status, and geographic location [US census region]), age, sex, setting, and quarter. All statistical analyses were conducted using R version 4.0.3 software (R Core Team 2020) and the R geepack package. *P* values <.05 were considered statistically significant.

## RESULTS

A total of 219 hospitals provided data during the 10-year study period (Supplementary Table 1). The number of hospitals contributing data over the years ranged from 77 in 2011 to 188 in 2019. Urban facilities accounted for 61.2% of facilities and the South US census region (51.1%) had the highest proportion of hospitals followed by the Midwest (18.7%), Northeast (16.4%), and West (13.7%) regions. Among the 7605 *S pneumoniae* isolates analyzed over the study period, 28.1% were from patients <2 years, 36.9% were from patients 2–4 years, and 35.1% were from patients 5–17 years (Table 1). Almost 90% of the isolates (6641 [87.3%]) were from noninvasive PD cases, primarily ENT (n = 3201) and respiratory (n = 2211) cultures, while the remainder (964 [12.7%]) were from IPD sources (872 from blood and 92 from CSF/neurological cultures).

**Table 1.** Distribution of 30-Day Nonduplicate *Streptococcus pneumoniae* Isolates by Invasive and Noninvasive Pneumococcal Disease Source in Pediatric Patients

Characteristic	Invasive PD		Noninvasive PD		Total	
	No.	(%)	No.	(%)	No.	(%)
Overall	964	(100)	6641	(100)	7605	(100)
Age, y						
<2	328	(34.0)	1807	(27.2)	2135	(28.1)
2–4	308	(32.0)	2495	(37.6)	2803	(36.9)
5–17	328	(34.0)	2339	(35.2)	2667	(35.1)
Sex						
Female	407	(42.2)	2904	(43.7)	3311	(43.5)
Male	557	(57.8)	3737	(56.3)	4294	(56.5)
Source						
Blood	872	(90.5)	…	…	872	(11.5)
Neurological	2	(0.2)	…	…	2	(<0.1)
CSF	90	(9.3)	…	…	90	(1.2)
Respiratory	…	…	2211	(33.3)	2211	(29.1)
ENT	…	…	3201	(48.2)	3201	(42.1)
Wound	…	…	934	(14.1)	934	(12.3)
Urine	…	…	204	(3.1)	204	(2.7)
Other	…	…	84	(1.3)	84	(1.1)
Onset						
Ambulatory	491	(50.9)	5158	(77.7)	5649	(74.3)
Admission	429	(44.5)	1214	(18.3)	1643	(21.6)
Hospital	44	(4.6)	269	(4.1)	313	(4.1)

Totals may not equal 100% due to rounding.

Abbreviations: CSF, cerebrospinal fluid; ENT, ear, nose, and throat; PD, pneumococcal disease.

### Antimicrobial Resistance in *S pneumoniae* Isolates

**Figure 1.**
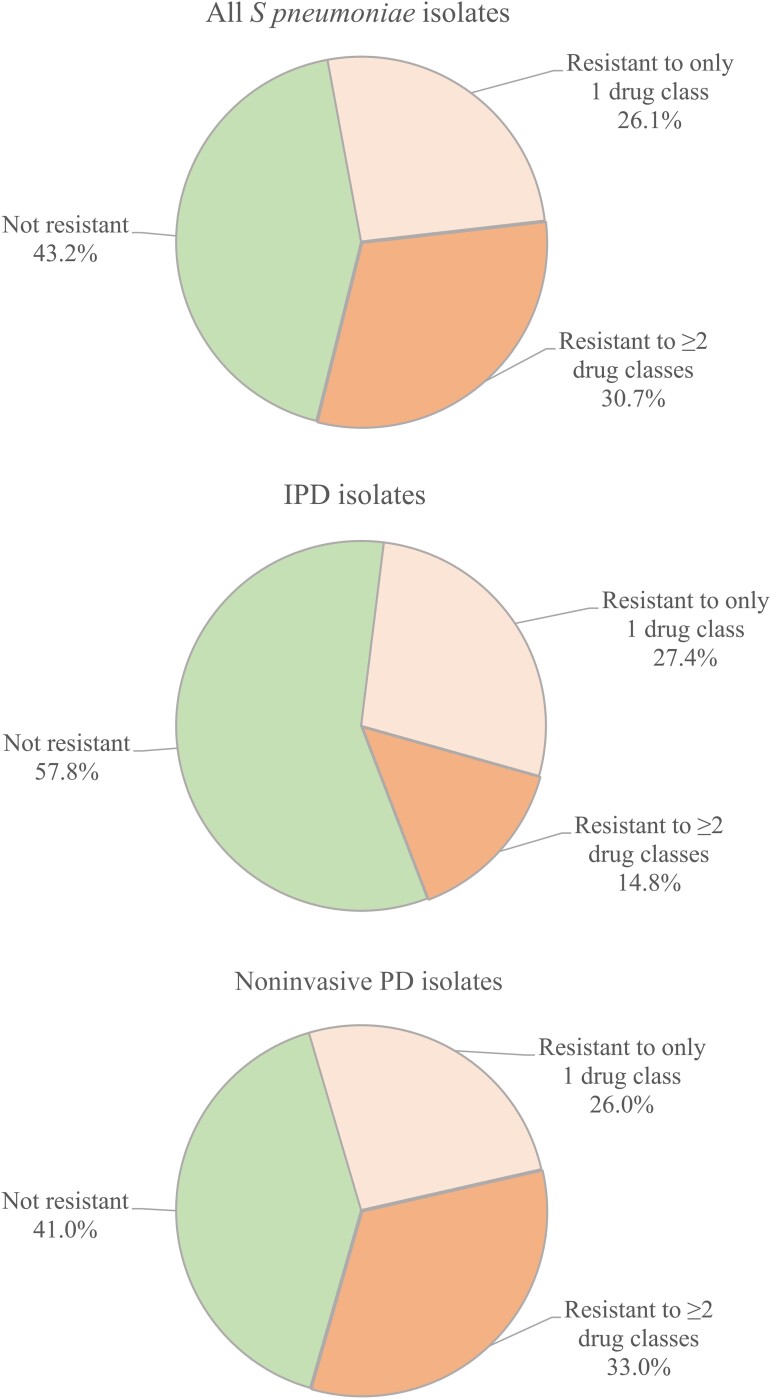
Resistance profiles of *Streptococcus pneumoniae* isolates by number of drugs based on observed data for all (N = 7605), invasive pneumococcal disease (IPD) (n = 964), and noninvasive pneumococcal disease (PD) (n = 6641) isolates.

The majority (56.8%) of isolates were resistant to at least 1 drug class (≥1 drug class) during the study period, and 30.7% of isolates were resistant to ≥2 drug classes (Figure 1; Table 2). There were too few isolates with resistance to ≥3 drug classes for meaningful analyses (n = 637).

**Table 2.** Observed Antimicrobial Resistance in *Streptococcus pneumoniae* Isolates in Children (January 2011–February 2020)

Antibiotic	Invasive PD (n = 964)	Noninvasive PD (n = 6641)	Total (N = 7605)
Resistance to ≥1 drug class	407 (42.2)	3915 (59.0)	4322 (56.8)
Resistance to ≥2 drug classes	143 (14.8)	2193 (33.0)	2336 (30.7)
Resistance by antibiotic class			
Macrolide	306 (31.7)	2731 (41.1)	3037 (39.9)
Penicillin	199 (20.6)	2813 (42.4)	3012 (39.6)
Tetracycline	72 (7.5)	761 (11.5)	833 (11.0)
ESC	34 (3.5)	483 (7.3)	517 (6.8)
Fluoroquinolone	4 (0.4)	29 (0.4)	33 (0.4)

Data are presented as No. (%). Totals may not equal 100% due to rounding.

Abbreviations: ESC, extended-spectrum cephalosporins; PD, pneumococcal disease.

Approximately 40% of isolates were resistant to macrolides (39.9%) or to penicillin (39.6%) (Table 2). Tetracycline resistance was observed in 11.0% of isolates, ESC resistance in 6.8%, and fluoroquinolone resistance in 0.4% of the isolates. The remainder of the analyses were focused on resistance to macrolides or penicillin, as tetracycline is generally not recommended in pediatric patients aged <8 years [14] and the proportions of isolates with ESC and fluoroquinolone resistance were low.

A higher proportion of noninvasive PD compared with IPD isolates was resistant to ≥1 drug class (59.0% vs 42.2%), ≥2 drug classes (33.0% vs 14.8%), macrolides (41.1% vs 31.7%), and penicillin (42.4% vs 20.6%) (Table 2). Multivariate models confirmed the statistical significance of these differences in adjusted analyses (all *P* < .001; Table 3).

**Table 3.** Adjusted *Streptococcus pneumoniae* Resistance Rates Overall and by Age and Culture Source

Characteristic	% Resistance to ≥1 Drug Class			% Resistance to ≥2 Drug Classes			% Macrolide-Resistant Isolates			% Penicillin-Resistant Isolates		
	IPD	Noninvasive PD	Total	IPD	Noninvasive PD	Total	IPD	Noninvasive PD	Total	IPD	Noninvasive PD	Total
Overall	41.7 (39.7–43.6)	58.4 (57.4–59.4)	56.1 (55.6–56.6)	15.1 (13.7–16.5)	34.7 (33.7–35.6)	31.6 (30.9–32.2)	32.9 (31.4–34.4)	47.5 (46.3–48.6)	45.1 (44.1–46.2)	18.9 (17.2–20.7)	41.9 (40.6–43.2)	40.2 (38.1–42.2)
Age, y, *P* value	.009	<.001	<.001	.161	<.001	<.001	.141	<.001	<.001	.068	<.001	<.001
<2	40.7 (38.9–42.5)	65.7 (64.0–67.3)	61.9 (60.8–63.1)	14.7 (12.7–16.6)	39.1 (37.2–40.9)	35.1 (33.6–36.5)	33.0 (31.0–35.0)	52.9 (51.2–54.6)	49.5 (48.5–50.5)	17.0 (15.9–18.1)	48.0 (46.5–49.6)	44.8 (43.6–46.1)
2–4	47.3 (45.4–49.3)	60.1 (58.6–61.5)	58.2 (57.3–59.2)	18.3 (16.2–20.3)	36.2 (34.6–37.7)	33.5 (32.2–34.7)	38.2 (36.1–40.4)	49.1 (47.6–50.6)	47.3 (46.4–48.2)	22.7 (21.4–23.9)	42.8 (41.4–44.3)	41.5 (40.3–42.7)
5–17	37.3 (35.5–39.1)	50.7 (49.2–52.1)	48.9 (48.0–49.8)	12.6 (10.6–14.6)	29.4 (27.9–31.0)	26.6 (25.4–27.9)	27.8 (25.8–29.8)	41.3 (39.8–42.7)	38.9 (37.8–40.1)	17.3 (16.2–19.3)	35.8 (34.4–37.3)	34.8 (33.6–35.9)
Source, *P* value	.012	.179	<.001	.028	.556	<.001	<.001	.002	<.001	.667	.844	<.001
Blood	42.4 (41.4–43.5)	…	47.0 (45.2–48.7)	15.7 (14.2–17.2)	…	18.1 (15.9–20.4)	34.6 (33.0–36.2)	…	37.7 (36.3–39.1)	18.7 (16.9–20.6)	…	20.3 (18.9–21.7)
CSF/neurological	34.3 (32.0–36.6)	…	38.6 (35.6–41.6)	9.7 (7.3–12.1)	…	12.4 (10.1–14.7)	16.8 (14.8–18.7)	…	21.9 (19.8–23.9)	20.6 (18.0–23.1)	…	21.0 (19.4–22.6)
Respiratory	…	59.7 (58.0–61.4)	59.2 (58.0–60.5)	…	34.7 (33.0–36.5)	34.3 (32.8–35.8)	…	49.7 (48.0–51.4)	49.1 (47.9–50.2)	…	42.1 (40.5–43.7)	42.0 (40.6–43.4)
ENT	…	57.5 (55.3–58.7)	56.9 (56.1–57.8)	…	34.6 (33.3–35.9)	33.9 (32.8–35.0)	…	45.9 (44.6–47.2)	44.9 (43.1–46.7)	…	41.8 (40.4–43.1)	41.8 (40.7–43.0)

Unless otherwise indicated, data are presented as estimated percentage resistance (95% confidence interval). Models were adjusted by age, sex, setting, quarter, hospital demographics, and United States census region. Additional variables are presented in Supplementary Table 2.

Abbreviations: CSF, cerebrospinal fluid; ENT, ear, nose, throat; IPD, invasive pneumococcal disease; PD, pneumococcal disease.

### Association of Demographic and Hospital Characteristics With *S pneumoniae* Resistance

In multivariate analyses of total *S pneumoniae* isolates, significant differences among age groups were observed for resistance to ≥1 drug class, ≥2 drug classes, macrolides, and penicillin (Table 3). The <2-year-old age group had the highest levels of resistance and the 5- to 17-year-old age group had the lowest. As mentioned above, resistance was higher in noninvasive PD (respiratory, ENT) than in IPD (blood, CSF/neurological) isolates. Other characteristics associated with higher rates of *S pneumoniae* resistance included hospital bed size (≥1 drug class), hospital-onset cultures (all drug categories), and geographic region (all drug categories) (Supplementary Table 2).

Noninvasive PD isolates showed similar age patterns to total isolates. Macrolide resistance rates were significantly higher for respiratory versus ENT isolates, but resistance rates for ≥1 drug class, ≥2 drug classes, and penicillin were similar for these sources. Other characteristics associated with higher resistance rates in noninvasive PD isolates included hospital bed size (≥2 drug classes and macrolides), hospital-onset cultures (all drug categories), urban/rural location (macrolides), teaching versus nonteaching hospitals (macrolides), and geographic region (all drug categories) (Supplementary Table 2).

In contrast to the age pattern observed for total and noninvasive PD isolates, for IPD isolates the 2 to 4-year-old age group had the highest levels of resistance to ≥1 drug class, ≥2 drug classes, macrolides, and penicillin (Table 3). Resistance to all evaluated drug categories was higher for blood versus CSF/neurological isolates. Other characteristics associated with higher resistance rates in IPD isolates included hospital bed size (≥1 drug class and macrolides), hospital-onset cultures (≥2 drug classes, macrolides, and penicillin), teaching versus nonteaching hospitals (≥1 drug class), and geographic region (all drug categories) (Supplementary Table 2).

### Trends in AMR Over Time

In adjusted analyses, *S pneumoniae* resistance to ≥1 drug class and ≥2 drug classes increased significantly from January 2011 to February 2020 (Table 4). The annual rate of increase was +0.9% per year for ≥1 drug class and +1.8% per year for ≥2 drug classes over this time period (*P* < .001 for both analyses). Noninvasive PD isolates showed similar increases (+0.6% per year for ≥1 drug class and +2.2% per year for ≥2 drug classes; *P* < .001 for both analyses). The rate of IPD isolates with resistance to ≥1 or ≥2 drug classes fluctuated during this time period, but trends were not significant.

**Table 4.** Model-Estimated Rates of Resistance and Annual Percentage Change in *Streptococcus pneumoniae* Resistance to ≥1 and ≥2 Drugs in Isolates Collected From US Children (January 2011–February 2020)

Year	Estimated % Resistance to ≥1 Drug Class			Estimated % Resistance to ≥2 Drug Classes			Estimated % Resistance to Macrolides			Estimated % Resistance to Penicillins		
	IPD	Noninvasive PD	Total	IPD	Noninvasive PD	Total	IPD	Noninvasive PD	Total	IPD	Noninvasive PD	Total
2011	40.1 (35.4–44.8)	56.7 (54.0–59.5)	54.3 (52.1–56.5)	20.2 (18.2–22.1)	29.5 (27.5–31.5)	27.9 (26.4–29.5)	26.8 (24.3–29.2)	25.5 (23.0–28.0)	25.6 (23.7–27.4)	26.8 (24.8–28.9)	48.7 (46.6–50.8)	47.4 (45.6–49.1)
2012	38.1 (33.2–43.0)	52.8 (51.0–55.7)	50.6 (48.3–53.0)	14.0 (11.2–16.9)	22.1 (19.9–24.2)	20.4 (18.6–22.1)	22.7 (20.2–25.2)	21.3 (18.7–24.0)	21.2 (19.2–23.1)	21.6 (19.6–23.6)	43.5 (41.3–45.4)	41.6 (39.8–43.5)
2013	43.5 (38.4–48.5)	52.8 (50.1–55.5)	51.3 (49.1–53.5)	13.4 (11.2–15.6)	21.1 (19.1–23.1)	19.5 (17.9–21.1)	36.9 (35.2–38.6)	24.7 (22.2–27.2)	25.8 (24.0–27.6)	17.6 (15.3–20.0)	41.4 (39.3–43.4)	39.4 (37.7–41.2)
2014	46.4 (42.5–50.4)	57.2 (54.3–60.0)	55.6 (53.3–58.0)	14.0 (11.2–16.9)	32.4 (30.2–34.6)	29.4 (27.6–31.1)	32.1 (30.6–33.7)	45.3 (42.7–47.9)	42.9 (40.9–44.8)	23.6 (20.5–26.7)	41.9 (39.8–44.0)	40.8 (39.0–42.6)
2015	36.2 (33.6–38.8)	64.6 (61.9–67.3)	60.3 (58.2–62.4)	13.9 (12.9–15.0)	44.6 (42.6–46.6)	39.5 (37.9–41.0)	28.2 (26.9–29.6)	63.7 (61.3–66.2)	57.6 (55.8–59.3)	16.8 (15.6–17.9)	45.8 (43.8–47.8)	43.3 (41.6–45.0)
2016	38.4 (35.5–41.3)	58.6 (56.2–61.0)	55.8 (53.8–57.7)	14.2 (12.8–15.6)	41.2 (39.5–42.9)	36.9 (35.2–38.6)	33.8 (32.1–35.5)	58.5 (56.2–60.7)	54.5 (52.9–56.0)	16.1 (14.6–17.5)	42.0 (40.1–43.9)	39.6 (38.0–41.2)
2017	42.6 (39.7–45.6)	61.0 (58.3–63.6)	58.3 (56.2–60.4)	19.1 (17.8–20.4)	40.9 (38.9–42.9)	37.4 (35.9–38.9)	37.6 (36.0–39.3)	61.1 (58.6–63.5)	57.1 (55.4–58.8)	19.4 (18.0–20.8)	38.9 (36.9–40.9)	37.9 (36.2–39.6)
2018	46.2 (42.6–49.8)	58.2 (56.1–60.3)	56.7 (54.2–59.2)	16.0 (14.1–17.9)	38.2 (36.7–37.7)	34.8 (32.9–36.7)	37.9 (36.5–39.2)	59.7 (57.0–62.5)	56.1 (54.0–58.1)	19.8 (17.7–21.8)	37.1 (34.9–39.3)	36.4 (34.5–38.3)
2019	43.0 (39.4–46.6)	61.4 (59.6–63.3)	58.5 (56.3–60.8)	9.7 (7.9–11.5)	39.8 (38.6–41.0)	35.0 (33.3–36.7)	35.6 (34.2–36.9)	61.8 (59.2–64.4)	57.4 (55.6–59.3)	11.6 (9.6–13.6)	37.8 (35.7–39.9)	35.4 (33.6–37.2)
2020 (Jan–Feb)	50.4 (47.6–53.2)	66.1 (63.0–69.1)	64.2 (60.9–67.5)	17.9 (15.4–20.4)	39.9 (37.3–42.4)	36.4 (33.7–38.9)	41.4 (39.3–43.5)	69.1 (65.7–72.5)	64.3 (61.8–67.8)	19.5 (15.6–23.4)	37.2 (34.1–39.3)	35.8 (32.2–39.3)
Annual % change	+0.05% (−.03 to .12)	+0.6% (.2–1.1)	+0.9% (.4–1.3)	−0.3% (−.9 to .3)	+2.2% (1.7–2.7)	+1.8% (1.4–2.3)	+1.4% (.3–2.5)	+5.7 (5.2–6.2)	+5.0 (4.5–5.5)	−1.0 (−1.9 to .2)	−1.1 (−1.6 to .6)	−1.1 (−1.5 to .7)
*P* value	.353	<.001	<.001	.480	<.001	<.001	.005	<.001	<.001	.006	<.001	<.001

Unless otherwise indicated, data are presented as estimated percentage resistance (95% confidence interval). Models were adjusted by age, sex, setting, quarter, hospital demographics, and United States census region. Abbreviation: IPD, invasive pneumococcal disease; PD, pneumococcal disease.

For total *S pneumoniae* isolates, resistance to macrolides increased (+5.0% per year, *P* < .001) and resistance to penicillin decreased (−1.1% per year, *P* < .001) (Table 4). The same pattern was observed for noninvasive PD (+5.7% per year for macrolides, *P* < .001; −1.1% for penicillin, *P* < .001) and IPD isolates (+1.4% per year for macrolides, *P* = .005; −1.0% for penicillin, *P* = .006).

## DISCUSSION

*Streptococcus pneumoniae* remains an important cause of pediatric infections despite marked decreases in disease incidence following introduction of PCVs. Effective antibiotics against pneumococci are available, but management of this pathogen can be complicated by AMR. As documented in this study, more than half (56.1%) of 7605 *S pneumoniae* isolates from US children were resistant to at least 1 drug class in the adjusted analyses, and there were persistently high levels of AMR, particularly to macrolides and penicillin, over the last decade. Multivariate analyses demonstrated significant increasing trends in the proportion of total and noninvasive PD isolates resistant to ≥1 drug class and ≥2 drug classes, and a significant increasing trend in macrolide-resistant isolates for both noninvasive PD and IPD sources. The magnitudes of increasing resistance to ≥2 drug classes (+1.8%/year) and macrolides (+5.0%/year) were particularly alarming. High and increasing *S pneumoniae* resistance to macrolides has also been documented in adults with PD [16, 17]. More encouraging results were observed with penicillin resistance, which showed a significant decreasing trend, although the approximately 35% resistance rate for total isolates in the most recent data (2019–2020) is still quite substantial. Our findings are consistent with a meta-analysis that reported high levels of penicillin and macrolide resistance in *S pneumoniae* in children globally [7].

A notable result from this study was higher resistance rates in noninvasive PD isolates compared with IPD isolates, regardless of the drug category evaluated. A similar observation has also been made in adults [17]. Although noninvasive PD cases are generally less severe, they contribute to widespread antibiotic use [18–20] and exert a heavy burden on the healthcare system, including an estimated 382 182 pneumonia hospitalizations and 93 million office visits for otitis media in US children between 1997 and 2019 [2]. It is likely that the observed resistance in pediatric noninvasive PD isolates is related to high levels of antibiotic use for outpatient respiratory infections, as has been suggested by some studies [21, 22]. We also found higher resistance rates in children under the age of 2 compared with older age groups for total and noninvasive PD isolates. This finding is of significant concern, as children <2 years of age are more vulnerable to *S pneumoniae* infections and deaths than older pediatric age groups [23, 24].

Drug-resistant *S pneumoniae* infections represent one of the few antibiotic-resistant threats that can be mitigated by vaccines [12]. Although US childhood PCV vaccination rates are generally high (82%–92%), lagging vaccination rates are observed in some populations, including uninsured children and children living below the poverty level, and coronavirus disease 2019–related disruptions in vaccination schedules have further exacerbated these disparities [25]. Pneumococcal vaccines reduce AMR by decreasing the frequency of *S pneumoniae* serotypes that are more likely to be antibiotic resistant [3, 7] and by reducing respiratory infections that result in antibiotic use [9]. Reductions in rates of PD are also associated with reductions in antimicrobial-resistant *S pneumoniae* caused by serotypes covered by vaccines [3]. However, there has been a recent increase in antimicrobial-resistant *S pneumoniae* due to serotypes not covered by 13-valent PCV (PCV13) [3].

Accordingly, another potential defense against antimicrobial-resistant pneumococcal infections is expanded-valency vaccines. New PCVs covering additional serotypes (15-valent PCV [PCV15] and 20-valent PCV [PCV20]) have recently been approved for adults in the US [26, 27], and PCV15 has also been approved for children in the US [27]. PCV15 and PCV20 cover several additional serotypes that contribute to resistant IPD [3]. Modeling studies indicate that these extended-valency vaccines have the potential to substantially reduce the burden of PD in US children [28, 29]. The profound effect of vaccines on disease burden is further expanded when economic evaluations of AMR reduction are included [30]. Both the World Health Organization and the US National Vaccine Advisory Committee have endorsed the use of vaccines to decrease levels of antibiotic-resistant bacteria [31, 32].

Although vaccines are a critical component of initiatives to combat AMR in *S pneumoniae*, effective antimicrobial stewardship efforts are also needed to combat resistance in *S pneumoniae* and other pathogens. Antibiotic overuse continues to be a problem that drives community AMR for *S pneumoniae* [21, 33]. The issue of inappropriate antibiotic use in US children has been well documented [19, 34–36]. Although antibiotics are not routinely recommended for pediatric outpatients with community-acquired pneumonia (CAP), which is often due to viral infections, 73.9% of US children aged 1–6 years with CAP between 2008 and 2015 were nonetheless prescribed antibiotics based on nationwide data [19]. Counter to current guidelines, macrolides are the most common antibiotic class prescribed for ambulatory CAP in children, with approximately 40% of pediatric patients receiving macrolide treatment [19, 34, 35]. This widespread usage is likely a contributor to the high (adjusted rate of 45.1%) and increasing macrolide resistance rate observed in our study and suggests that targeted antimicrobial stewardship efforts may be required to reduce AMR in *S pneumoniae*. Both inpatient and outpatient stewardship programs are key to achieving this goal [37, 38]. Our data may also be useful in the critical examination of guidelines for treating common respiratory diseases [39].

Our study has limitations, including the use of facility reports to assess AMR, which may have influenced reported resistance rates and contributed to a lack of consistency in resistance thresholds across different facilities. This consideration is particularly relevant for penicillin resistance rates. In 2013, the Clinical and Laboratory Standards Institute (CLSI) increased the nonmeningitis nonsusceptibility breakpoint for parenteral penicillin from an MIC of >0.06 mg/L to >2 mg/L [40]. Use of the lower breakpoint could cause inflated rates of resistance and may have influenced trends in resistance over time. In a recent analysis of US *S pneumoniae* isolates (n = 7901), primarily (88.5%) from respiratory tract infections, penicillin-nonsusceptible rates were 38.8% using the meningitis/oral penicillin breakpoint (>0.06 mg/L) and 6.2% using the parenteral penicillin breakpoint (>2 mg/L) [41]. CLSI changes can take many years to be fully adopted at the clinical laboratory level [42]; a recent survey of 1490 US clinical laboratories reported that between 30% and 62% of laboratories used obsolete breakpoints for at least 1 of the pathogens evaluated, although *S pneumoniae* was not included in this study [43]. It is therefore possible that delayed adoption of this CLSI change increased the percentage of penicillin-resistant *S pneumoniae* isolates reported by facilities, which could explain the discrepancy between our data and lower numbers (3.6%) reported by the CDC, which utilizes a central laboratory for resistance rate assessment [24]. Changing resistance breakpoints may also have influenced the decreased resistance to penicillin observed in our study. It is important to note, however, that the data presented here reflect the susceptibility data on isolates available to clinicians during daily management of their patients. Other potential limitations of our study include lack of information on associated infections; our analyses were based solely on culture-positive isolates, and the presence of symptomatic PD was not confirmed. Selection bias, which can affect all microbiologic surveillance studies, may increase estimates of resistance due to a higher likelihood of testing more severely ill patients. We did not evaluate *S pneumoniae* serotypes or the pneumococcal vaccination status of patients. These data would be valuable additions to a future analysis.

In conclusion, our data document high levels of resistance to macrolides and penicillin in *S pneumoniae* isolates among US children with IPD and noninvasive PD infections. These data may help inform initial treatment decisions for pediatric outpatients and inpatients with suspected pneumococcal infections. The rates of resistance to ≥1 drug class,≥2 drug classes, and macrolides have increased in pediatric *S pneumoniae* isolates over the past 10 years, despite overall disease reductions attributable to vaccines. Addressing the high and increasing rates of *S pneumoniae* resistance may require PCVs with expanded serotype coverage and targeted antimicrobial stewardship efforts [3, 37, 38].

## Supplementary Material

ofad098_Supplementary_Data

## Supplementary Data

Supplementary materials are available at *Open Forum Infectious Diseases* online. Consisting of data provided by the authors to benefit the reader, the posted materials are not copyedited and are the sole responsibility of the authors, so questions or comments should be addressed to the corresponding author.

## Notes

***Acknowledgments***. We thank Sharon L. Cross, PhD, Fusion MD Medical Science Network (Montreal, Canada), for providing manuscript support with funding from Becton, Dickinson & Company.

***Financial support***. This work was funded by a grant from Merck Sharp & Dohme LLC (MSD), a subsidiary of Merck & Co, Inc, to Becton, Dickinson & Company (BD). Medical writing was supported by BD.
