# Supplementary material for: Trends in Streptococcus pneumoniae Antimicrobial Resistance in US Children: A Multicenter Evaluation
Source: Open Forum Infect Dis. 2023 Mar 7;10(3):ofad098. doi: 10.1093/ofid/ofad098 (PMC10034583; doi:10.1093/ofid/ofad098)
Supplement: ofad098_Supplementary_Data [file ofad098_supplementary_data.zip › Mohanty_Suppl-Table-1.docx]

**Trends in *Streptococcus pneumoniae* Antimicrobial Resistance in US Children:**

**A Multicenter Evaluation**

Salini Mohanty, Kirsten Feemster, Kalvin C. Yu, Janet A. Watts, Vikas Gupta

**SUPPLEMENTAL MATERIAL**

**Supplementary Table 1. Hospital Demographics**

| **Characteristics** | **Number of Hospitals** | |
| --- | --- | --- |
|  | **n** | **%** |
| **Total** | 219 | 100.0 |
| **Year**^a^ |  |  |
| 2011 | 77 | 35.2 |
| 2012 | 85 | 38.8 |
| 2013 | 95 | 43.4 |
| 2014 | 103 | 47.0 |
| 2015 | 136 | 62.1 |
| 2016 | 144 | 65.8 |
| 2017 | 160 | 73.1 |
| 2018 | 176 | 80.4 |
| 2019 | 188 | 85.8 |
| 2020 (Q1) | 159 | 72.6 |
| **Bed size** |  |  |
| Less than 100 | 52 | 23.7 |
| 100-300 | 104 | 47.5 |
| Greater than 300 | 63 | 28.8 |
| **Urban/rural** |  |  |
| Rural | 85 | 38.8 |
| Urban | 134 | 61.2 |
| **Teaching status** |  |  |
| Non-teaching | 137 | 62.6 |
| Teaching | 82 | 37.4 |
| **US census regions**^b^ |  |  |
| South | 112 | 51.1 |
| Midwest | 41 | 18.7 |
| Northeast | 36 | 16.4 |
| West | 30 | 13.7 |

^a^ The number of hospitals varied by year; the total of 219 reflects all facilities that contributed data in any year.

^b^States included in each region can be found at <https://www2.census.gov/geo/pdfs/maps-data/maps/reference/us_regdiv.pdf>
